# Serum insulin-like growth factor-binding protein 2 levels as an indicator for disease severity in enterohemorrhagic *Escherichia coli* induced hemolytic uremic syndrome

**DOI:** 10.1080/0886022X.2021.1885445

**Published:** 2021-03-01

**Authors:** Yuko Tasaki, Natsumi Inoue, Masaki Shimizu, Naotoshi Sugimoto, Sayaka Ishikawa, Mao Mizuta, Tadafumi Yokoyama, Mondo Kuroda, Kazuhide Ohta, Akihiro Yachie, Taizo Wada

**Affiliations:** a Department of Pediatrics, School of Medicine, Institute of Medical, Pharmaceutical, and Health Sciences, Kanazawa University, Kanazawa, Japan; b Department of Child Health and Development, Graduate School of Medical and Dental Sciences, Tokyo Medical and Dental University, Tokyo, Japan; c Department of Physiology, School of Medicine, Institute of Medical, Pharmaceutical, and Health Sciences, Kanazawa University, Kanazawa, Japan; d Department of Pediatrics, Fukui Saiseikai Hospital, Fukui, Japan; e Department of Pediatrics, National Hospital Organization, Kanazawa Medical Center, Kanazawa, Japan; f Division of Medical Safety, Kanazawa University Hospital, Kanazawa, Japan

**Keywords:** Insulin-like growth factor-binding protein 2, enterohemorrhagic *Escherichia coli*, hemolytic uremic syndrome, biomarker

## Abstract

**Background:**

Insulin-like growth factor-binding protein (IGFBP) 2 plays an important role in the regulation of cell adhesion, migration, growth, and apoptosis. This study aimed to investigate the clinical significance of serum IGFBP2 as a biomarker for disease activity and severity in hemolytic uremic syndrome (HUS) induced by enterohemorrhagic *Escherichia coli* (EHEC).

**Methods:**

IGFBP2 production by human renal glomerular endothelial cells (RGECs) after exposure to Shiga toxin 2 (Stx-2) was investigated *in vitro*. Serum IGFBP2 levels in blood samples obtained from 22 patients with HUS and 10 healthy controls (HCs) were quantified using an enzyme-linked immunosorbent assay. The results were compared to the clinical features of HUS and serum tau and cytokine levels.

**Results:**

Stx-2 induced the production of IGFBP2 in RGECs in a dose-dependent manner. Serum IGFBP2 levels were significantly higher in patients with HUS than in HCs and correlated with disease severity. Additionally, serum IGFBP2 levels were significantly higher in patients with encephalopathy than in those without encephalopathy. A serum IGFBP2 level above 3585 pg/mL was associated with a high risk of encephalopathy. Furthermore, serum IGFBP2 levels significantly correlated with serum levels of tau and inflammatory cytokines associated with the development of HUS.

**Conclusions:**

Correlation of serum IGFBP2 level with disease activity in patients with HUS suggests that IGFBP2 may be considered as a possible indicator for disease activity and severity in HUS. Larger studies and additional experiments using various cells in central nervous system should elucidate the true value of IGFBP2 as a clinical diagnostic marker.

**Abbreviations:**

IGFBP: insulin-like growth factor-binding protein; HUS: hemolytic uremic syndrome; EHEC: enterohemorrhagic Escherichia coli; RGECs: renal glomerular endothelial cells; STx-2: Shiga toxin 2; HCs: healthy controls; LPS: lipopolysaccharide; ROC: receiver operating characteristic; sTNFR: soluble tumor necrosis factor receptor.

## Introduction

Hemolytic uremic syndrome (HUS), a life-threating complication of infection with enterohemorrhagic *Escherichia coli* (EHEC) infection that produces Shiga toxin (Stx) is clinically characterized by microangiopathic hemolytic anemia, thrombocytopenia, and acute kidney injury [1,2]. Neurologic complications including acute encephalopathy, which are observed in approximately 20% of patients with HUS, are associated with high morbidity and mortality [3,4].

Previous studies have demonstrated that laboratory markers including white blood cell count and serum levels of alanine aminotransferase, sodium, and total protein are useful for predicting disease severity in patients with HUS [5,6]. Furthermore, recent studies have reported that serum cytokine levels predict the severity of HUS [7–10]. In a previous study, we have identified that five serum biomarkers, including insulin-like growth factor (IGF)-binding protein (IGFBP) 2, could predict disease severity in patients with HUS [10].

IGFBP2, which belongs to the IGFBP family, binds to IGFs with high affinity. IGFBP2 regulates the activity of IGFs [11]; in addition, it has IGF-independent roles in metabolism [11]. Previous studies reported that serum IGFBP2 levels were significantly elevated in patients with malignancies [12], rheumatic diseases [13–15], and kidney diseases [16] and that serum IGFBP2 levels were closely related with disease severity. However, the role of serum IGFBP2 in the pathogenesis of HUS and its causal relationship with disease activity are not clear.

In the present study, we measured serum IGFBP2 levels in patients with HUS and assessed the correlation of serum IGFBP2 levels with measures of disease activity and severity, with the aim to elucidate its clinical relevance in these patients.

## Materials and methods

### Patients and samples

Serum samples were collected from 22 patients with HUS at the time of HUS diagnosis and 10 healthy controls (HCs). EHEC infection was diagnosed with microbiological identification. HUS diagnosis was based on the presence of thrombocytopenia (platelet count <150,000/mm^3^), hemolytic anemia with schistocytes, and acute kidney injury (increased creatinine level ≥50% above the age-related baseline) [17]. Acute encephalopathy was defined as the presence of neurological symptoms accompanied with pathological findings on magnetic resonance imaging. In the present study, the patients were classified into three groups; mild group (*n* = 7), who were not treated with dialysis, severe group (*n* = 5), who presented anuria requiring treatment with dialysis, and encephalopathy group (*n* = 10). The clinical characteristics of patients with HUS included in the study are shown in Table 1.

**Table 1.** Clinical characteristics of patients with EHEC-induced HUS.

Number of patients	22
Age (years) (mean, IQR)	7.5 (1–26)
Sex (M/F)	(8/14)
Type of EHEC	O157; 8, O111; 14
Severity	
Mild	7
Severe	5
Encephalopathy	10

EHEC: enterohemorrhagic *Escherichia coli*.

The patient samples in six HUS patients were obtained both during hemorrhagic colitis, i.e., the pre-HUS phase, and at the time of HUS diagnosis. Serum was separated from blood samples, divided into aliquots, and stored at −80 °C until further processing.

This study was approved by the Institutional Review Board of the Kanazawa University (approval number: 1051), and all specimens were used after receiving informed patient consent from the patients and/or their legal guardians.

### Cell culture

Human renal glomerular endothelial cells (RGECs) were cultured in endothelial cell media (ScienCell Research Laboratories, Carlsbad, CA) at 37 °C with 5% CO_2_. Briefly, 96-well plates were precoated with 7.5 μg/mL fibronectin (ScienCell Research Laboratories, Carlsbad, CA) for two hours at room temperature and washed once with phosphate-buffered saline without calcium and magnesium. Next, RGECs were seeded into 96-well plates at a density of 4 × 10^4^ cells/well. After 24 h of incubation, the cells were treated with 30 μg/mL lipopolysaccharide (LPS) and/or Shiga toxin 2 (Stx-2) (0.1, 1.0, or 10 ng/mL) (Nacalai Tesque, Kyoto, Japan) for 48 h. Subsequently, culture supernatants were collected, and stored at −80 °C until further processing.

### Quantification of IGFBP2, tau, and cytokine levels in sera and culture supernatants

Concentrations of IGFBP2, tau, neopterin, IL-6, sTNFR-I, and sTNFR-II levels were measured with enzyme-linked immunosorbent assays according to the manufacturer’s instructions (IGFBP2, RayBiotech, Norcross, GA; tau, Invitrogen, Camarillo, CA; neopterin, IBL, Hamburg, Germany; IL-6, sTNFR-I, and sTNFR-II, R&D Systems, Minneapolis, MN). Serum IGFBP2, neopterin, IL-6, sTNFR-I, and sTNFR-II levels were measured in all 22 patients. Serum tau levels were determined in 21 patients, because serum sample from one patient in mild group was not insufficient for the measurement.

### Statistical analysis

Statistical analysis was performed using GraphPad Prism 7 software (GraphPad, San Diego, CA). Multiple group comparisons of IGFBP2 levels in sera and culture supernatants were performed using the Kruskal–Wallis test with Dunn's multiple comparison test. Within-group comparisons were performed using paired Student’s *t*-test. Receiver operating characteristic (ROC) analysis was performed to determine the cutoff level, sensitivity and specificity. Correlation analysis was performed using Spearman’s rank correlation coefficient. A *p* value of <.05 indicated a statistically significant difference in all analyses.

## Results

### Stx-2 and LPS induce IGFBP2 production in RGECs

**Figure 1.**
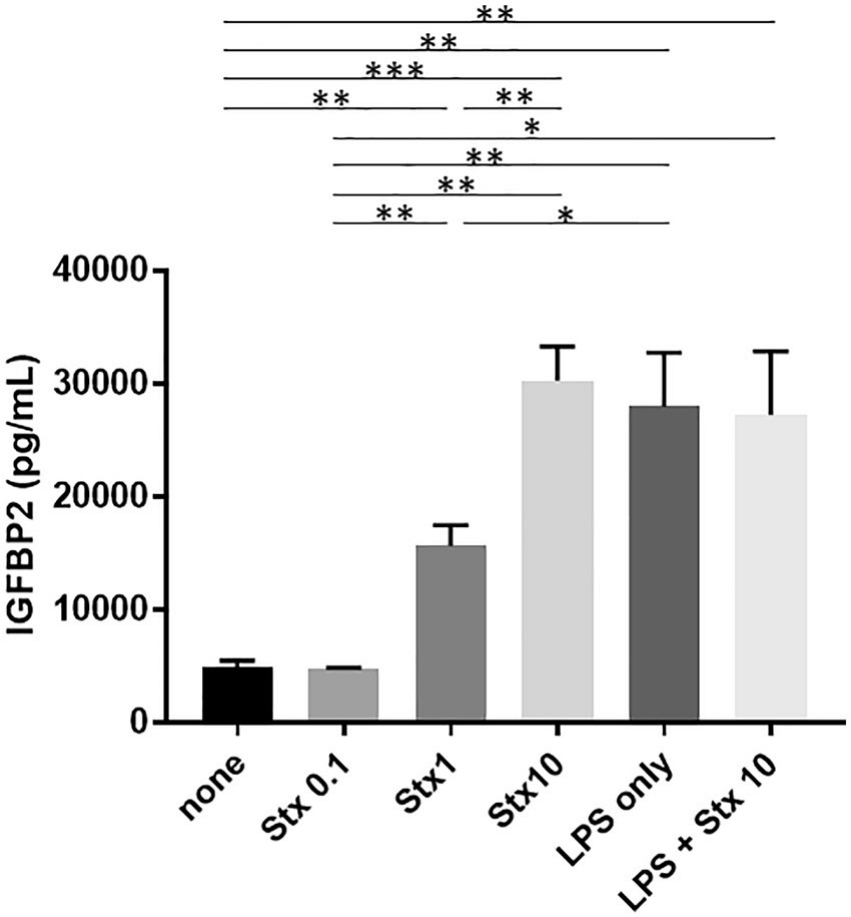
IGFBP2 production is induced by Stx-2 and LPS in RGECs. Data presented as mean (±standard error of the mean) of four experiments. Stx-2 dose-dependently induced the production of IGFBP2 in RGECs. LPS also induced the production of IGFBP2 in RGECs. Of note, there is no synergistic effect of STx2 on LPS-induced IGFBP2 production. *p<.05, **p<.01, and ***p<.001. LPS: lipopolysaccharide; RGEC: renal glomerular endothelial cell; Stx-2: Shiga toxin 2.

As shown in Figure 1, Stx-2 induced the production of IGFBP2 in RGECs in a dose-dependent manner. Furthermore, LPS also induced the production of IGFBP2 in RGECs. However, there was no synergistic effect of Stx-2 on LPS-mediated IGFBP2 production.

### Increased serum IGFBP2 levels in patients with EHEC-HUS

**Figure 2.**
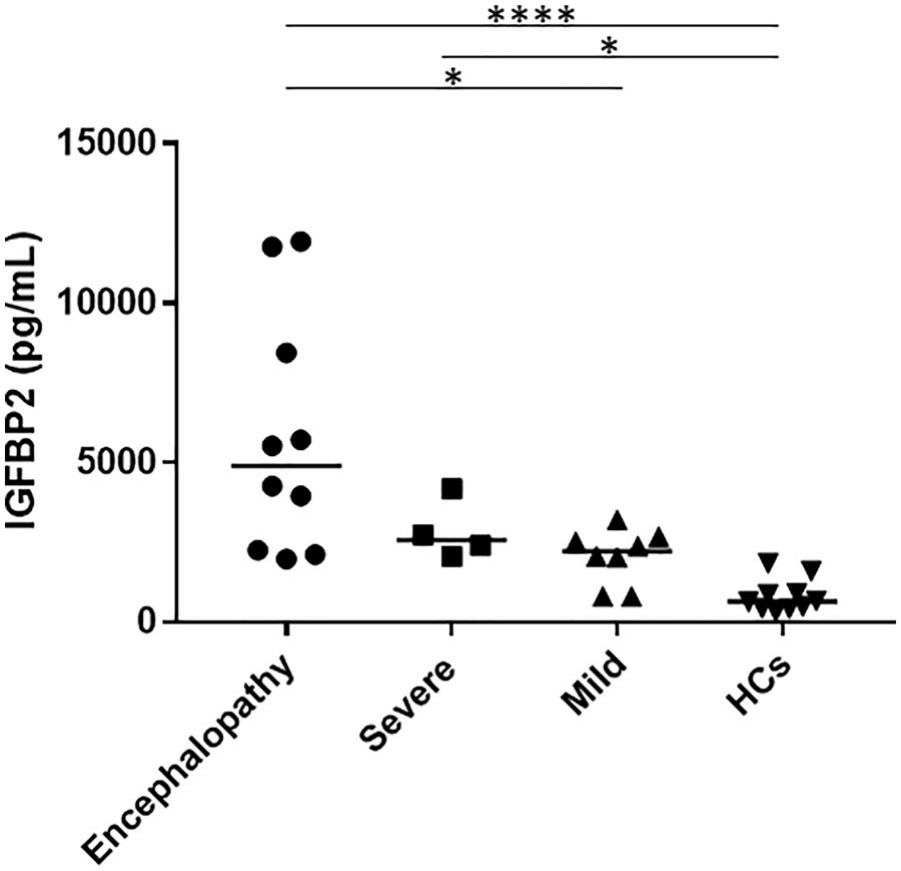
Serum IGFBP2 levels in patients with HUS. Bars represent median values. *p<.05 and ****p<.0001. HCs: healthy controls; IGFBP2: insulin-like growth factor-binding protein 2.

As shown in Figure 2, during the HUS phase, the median (range) serum IGFBP2 levels were significantly higher in patients with encephalopathy (4899 [1977–11,926] pg/mL) (*p*<.0001) and in those with severe HUS (2564 [2061–4180] pg/mL) (*p*<.05) compared with the HCs (649 [320–1840] pg/mL), whereas there was no significant difference in the serum IGFBP2 levels between the patients with mild HUS (2224 [802–3212] pg/mL) and HCs. Among the patients with HUS, serum IGFBP2 levels were significantly higher in those with encephalopathy than in those with mild HUS (*p*<.05). In contrast, there was no significant difference between the patients with severe HUS and those with mild HUS or between the patients with severe HUS and those with encephalopathy.

### Serum IGFBP2 levels as a biomarker for disease activity in HUS patients with encephalopathy

Serum IGFBP2 levels were significantly higher in patients with encephalopathy than in those without encephalopathy, which included those with severe and mild group (median, 2395 pg/mL; range, 802–4180 pg/mL) (*p*<.05). The ROC curve analysis indicated that the area under the curve and the optimal serum IGFBP2 cutoff level were 0.7917 and 3585 pg/mL for association with encephalopathy had a sensitivity of 91.7% and a specificity of 70%. Based on this serum IGFBP2 cutoff level, the odds ratio for HUS-related encephalopathy was 25.7 (95% confidence interval, 2.4–307.3), and the likelihood ratio was 3.056.

**Figure 3.**
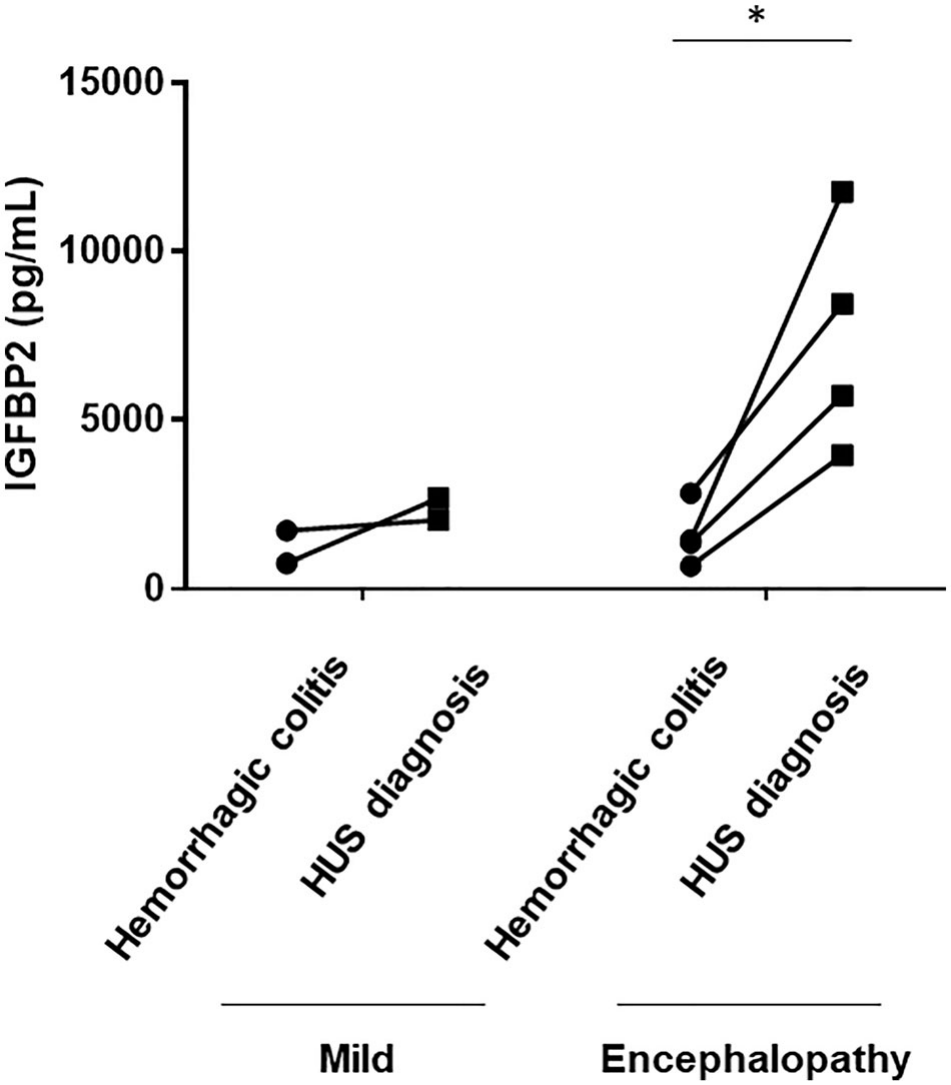
Changes in serum IGFBP2 levels in patients with HUS. Changes in serum IGFBP2 levels from the hemorrhagic colitis phase to the time of HUS diagnosis are shown. Serum IGFBP2 levels in encephalopathy group showed a significant and sharp increase with the progression of HUS, whereas no increase in serum IGFBP2 levels was observed in mild group. *p<.05.

Serum IGFBP2 levels were measured during the pre-HUS phase in six patients with HUS, including two patients with mild group and four patients with encephalopathy group. As shown in Figure 3, the serum IGFBP2 levels in patients with encephalopathy showed a significant and sharp increase with the progression of HUS (*p*<.05) whereas there was no increase in serum IGFBP2 levels with the progression of disease in patients with mild HUS.

### Correlation of serum IGFBP2 level with tau and proinflammatory cytokine levels in patients with HUS

**Figure 4.**
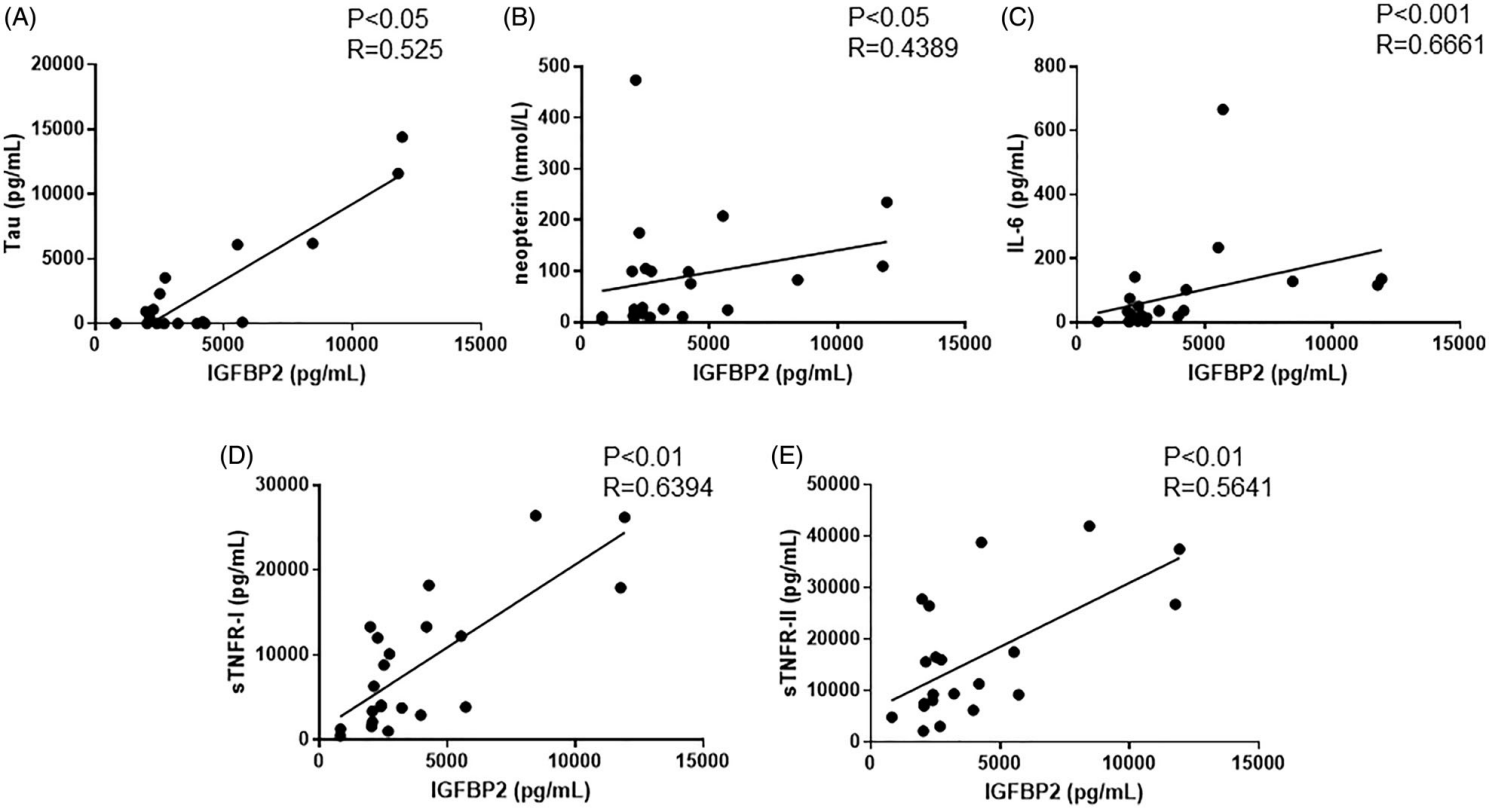
Correlations between serum IGFBP levels and serum tau and cytokine levels in patients with EHEC-HUS. (A) Tau (*n* = 21), (B) neopterin (*n* = 22), (C) interleukin 6 (IL-6; *n* = 22), (D) soluble tumor necrosis factor receptor (sTNFR) I (*n* = 22), and (E) sTNFR-II (*n* = 22).

As previously reported, serum levels of tau and proinflammatory cytokines, including neopterin, IL-6, sTNFR-I, and sTNFR-II, are indicators of disease activity in HUS. Therefore, we assessed the correlation of serum IGFBP2 levels with the serum levels of these cytokines. As shown in Figure 4, serum IGFBP2 levels positively correlated with tau (*p*< .05), neopterin (*p*< .05), IL-6 (*p*< .001), sTNFR-I (*p*< .01), and sTNFR-II (*p*< .01).

## Discussion

IGFBP2, a member of the IGFBP family proteins, plays an important role in regulating cell adhesion, migration, growth, and apoptosis through IGF-dependent and IGF-independent mechanisms [11]. Previous reports have demonstrated that serum IGFBP2, the 2nd most abundant IGFBP in serum [12], is a useful biomarker for assessing disease activity in various clinical conditions [12–16]. In the present study, we demonstrated for the first time that serum IGFBP2 levels in patients with HUS were markedly elevated during the active phase and that serum IGFBP2 level correlated with several measures of disease severity. Furthermore, serum IGFBP2 level was useful in predicting the development of encephalopathy. These findings suggest that IGFBP2 may have an important role in the pathogenesis of HUS and encephalopathy in particular. Furthermore, these results implicate serum IGFBP2 level as a potential marker of disease activity in patients with HUS and acute encephalopathy.

Previous studies reported that serum IGFBP2 levels were elevated in patients with kidney diseases including lupus nephritis [14,15] and diabetic nephropathy [16]. Furthermore, the glomerular IGFBP2 expression was shown to be increased in animal models of anti-glomerular basement membrane glomerulonephritis [18] and MRL/lpr lupus [19]. In the present study, the serum IGFBP2 levels were significantly elevated in patients with severe HUS compared with the HCs. Furthermore, Stx-2 and LPS induced IGFBP2 production in RGECs. These findings suggest that an increase in serum IGFBP2 levels in HUS might be due in part to kidney injury. Larger studies including the assessment of IGFBP2 localization and expression in kidney should provide more definitive evidence for the value of IGFBP2 as a clinical diagnostic marker for various kidney diseases.

In brain, IGFBP2 is expressed in olfactory bulbs, cortex, hippocampus, cerebellum, and amygdala [11]. IGFBP2 is also expressed in thalamus, which is frequently involved in EHEC-HUS [11,20]. *IGFBP2* mRNA is also detected in neurons and astrocytes as well as in meninges and blood vessels [11]. Intravenous injection of *E. coli* endotoxin in healthy humans elicits an increase in plasma IGFBP2 levels over several hours [21,22], whereas *Igfbp2* mRNA shows a dramatic increase 24–48 h after the injection of ciliary neurotrophic factor or IL-1β in an animal central nervous system injury model [23]. In the present study, serum IGFBP2 levels were significantly higher in patients with encephalopathy than in those without encephalopathy. Furthermore, serum IGFBP2 level was closely related to serum tau levels. Tau, a microtubule-associated protein localized in neurons and oligodendrocytes, is necessary for cytoskeletal structure and axonal transport. We previously reported that serum tau level was useful for in predicting and assessing disease activity in patients with EHEC encephalopathy [24]. These findings indicate that serum IGFBP2 levels might reflect disease activity in patients with encephalopathy associated with HUS. Specifically, our analyses suggest that patients with serum IGFBP2 levels above 3585 pg/mL were at the high risk for encephalopathy.

In the present study, we also found that serum IGFBP2 level showed a positive correlation with serum cytokine levels. Previous reports showed that tumor necrosis factor α, IL-6, interferon γ, and IL-1β could enhance the expression of IGFBP2 in astrocytes and microglia [25,26]. Although its role in the pathogenesis of HUS remains unknown, IGFBP2 may protect cells from cytokine release. In an animal model of brain injury, *Igfbp2* mRNA expression is markedly increased around the site of injury [27]. IGFBP2 protects cells from apoptosis in cooperation with the B-cell lymphoma 2 family of proteins [28]. Altogether, these various lines of evidence suggest that IGFBP2 might promote neuronal survival by preventing apoptosis.

A major limitation of this study was the small number of patients with HUS. Additionally, we did not investigate IGFBP2 production after the exposure of astrocytes and microglia to Stx-2. Larger studies and additional experiments using various cells in central nervous system should elucidate the true value of IGFBP2 as a clinical diagnostic marker.

## Conclusions

In conclusion, the present study results suggest that IGFBP2 plays an important role in the complex pathogenesis of HUS. Serum IGFBP2 levels may be considered as a possible indicator for disease severity in HUS.

## References

[1] Zoja C, Buelli S, Morigi M. Shiga toxin-associated hemolytic uremic syndrome: pathophysiology of endothelial dysfunction. Pediatr Nephrol. 2010;25(11):2231–2240. 20424866 10.1007/s00467-010-1522-1

[2] Scheiring J, Andreoli SP, Zimmerhackl LB. Treatment and outcome of Shiga-toxin-associated hemolytic uremic syndrome (HUS). Pediatr Nephrol. 2008;23(10):1749–1760. 18704506 10.1007/s00467-008-0935-6 PMC6901419

[3] Hahn JS, Havens PL, Higgins JJ, et al. Neurological complications of hemolytic-uremic syndrome. J Child Neurol. 1989;4(2):108–113. 2715605 10.1177/088307388900400206

[4] Siegler RL. Spectrum of extrarenal involvement in postdiarrheal hemolytic-uremic syndrome. J Pediatr. 1994;125(4):511–518. 7931868 10.1016/s0022-3476(94)70001-x

[5] Kamioka I, Yoshiya K, Satomura K, et al. Risk factors for developing severe clinical course in HUS patients: a national survey in Japan. Pediatr Int. 2008;50(4):441–446. 19143964 10.1111/j.1442-200X.2008.02605.x

[6] Buteau C, Proulx F, Chaibou M, et al. Leukocytosis in children with *Escherichia coli* O157:H7 enteritis developing the hemolytic-uremic syndrome. Pediatr Infect Dis J. 2000;19:642–647. 10917223 10.1097/00006454-200007000-00012

[7] Shiraishi M, Ichiyama T, Matsushige T, et al. Soluble tumor necrosis factor receptor 1 and tissue inhibitor of metalloproteinase-1 in hemolytic uremic syndrome with encephalopathy. J Neuroimmunol. 2008;196(1–2):147–152. 18410971 10.1016/j.jneuroim.2008.02.012

[8] Shimizu M, Kuroda M, Sakashita N, et al. Cytokine profiles of patients with enterohemorrhagic *Escherichia coli* O111-induced hemolytic-uremic syndrome. Cytokine. 2012;60(3):694–700. 22929411 10.1016/j.cyto.2012.07.038

[9] Shimizu M, Inoue N, Kuroda M, et al. Angiopoietin-1 and -2 as markers for disease severity in hemolytic uremic syndrome induced by enterohemorrhagic *Escherichia coli*. Clin Exp Nephrol. 2017;21(1):76–82. 26945868 10.1007/s10157-016-1254-z

[10] Shimizu M, Kuroda M, Inoue N, et al. Extensive serum biomarker analysis in patients with enterohemorrhagic *Escherichia coli* O111-induced hemolytic-uremic syndrome. Cytokine. 2014;66(1):1–6. 24548418 10.1016/j.cyto.2013.12.005

[11] Khan S. IGFBP-2 signaling in the brain: from brain development to higher order brain functions. Front Endocrinol (Lausanne). 2019;10:822. 31824433 10.3389/fendo.2019.00822 PMC6883226

[12] Russo VC, Azar WJ, Yau SW, et al. IGFBP-2: the dark horse in metabolism and cancer. Cytokine Growth Factor Rev. 2015;26(3):329–346. 25544066 10.1016/j.cytogfr.2014.12.001

[13] Neidel J. Changes in systemic levels of insulin-like growth factors and their binding proteins in patients with rheumatoid arthritis. Clin Exp Rheumatol. 2001;19:81–84. 11247331

[14] Ding H, Kharboutli M, Saxena R, et al. Insulin-like growth factor binding protein-2 as a novel biomarker for disease activity and renal pathology changes in lupus nephritis. Clin Exp Immunol. 2016;184(1):11–18. 26616478 10.1111/cei.12743 PMC4778092

[15] Mok CC, Ding HH, Kharboutli M, et al. Axl, ferritin, insulin-like growth factor binding protein 2, and tumor necrosis factor receptor type II as biomarkers in systemic lupus erythematosus. Arthritis Care Res (Hoboken). 2016;68(9):1303–1309. 26749069 10.1002/acr.22835 PMC5441892

[16] Narayanan RP, Fu B, Heald AH, et al. IGFBP2 is a biomarker for predicting longitudinal deterioration in renal function in type 2 diabetes. Endocr Connect. 2012;1(2):95–102. 23781310 10.1530/EC-12-0053 PMC3681324

[17] Buder K, Latal B, Nef S, et al. Neurodevelopmental long-term outcome in children after hemolytic uremic syndrome. Pediatr Nephrol. 2015;30(3):503–513. 25234636 10.1007/s00467-014-2950-0

[18] Fujinaka H, Katsuyama K, Yamamoto K, et al. Expression and localization of insulin-like growth factor binding proteins in normal and proteinuric kidney glomeruli. Nephrology (Carlton). 2010;15(7):700–709. 21040165 10.1111/j.1440-1797.2010.01285.x

[19] Mohammed JA, Mok AY, Parbtani A, et al. Increased expression of insulin-like growth factors in progressive glomerulonephritis of the MRL/lpr mouse. Lupus. 2003;12(8):584–590. 12945716 10.1191/0961203303lu422oa

[20] Takanashi J, Taneichi H, Misaki T, et al. Clinical and radiologic features of encephalopathy during 2011 *E coli* O111 outbreak in Japan. Neurology. 2014;82(7):564–572. 24443449 10.1212/WNL.0000000000000120

[21] Benbassat CA, Lazarus DD, Cichy SB, et al. Interleukin-1 alpha (IL-1 alpha) and tumor necrosis factor alpha (TNF alpha) regulate insulin-like growth factor binding protein-1 (IGFBP-1) levels and mRNA abundance in vivo and in vitro. Horm Metab Res. 1999;31(2–3):209–215. 10226804 10.1055/s-2007-978721

[22] Lang CH, Pollard V, Fan J, et al. Acute alterations in growth hormone-insulin-like growth factor axis in humans injected with endotoxin. Am J Physiol. 1997;273(1 Pt 2):R371–R378. 9249574 10.1152/ajpregu.1997.273.1.R371

[23] Wood TL, O'Donnell SL, Levison SW. Cytokines regulate IGF binding proteins in the CNS. Prog Growth Factor Res. 1995;6(2–4):181–187. 8817660 10.1016/0955-2235(95)00035-6

[24] Kuroda M, Shimizu M, Inoue N, et al. Serum tau protein as a marker of disease activity in enterohemorrhagic *Escherichia coli* O111-induced hemolytic uremic syndrome. Neurochem Int. 2015;85–86:24–30. 10.1016/j.neuint.2015.04.003 25895963

[25] Chesik D, De Keyser J, Wilczak N. Insulin-like growth factor binding protein-2 as a regulator of IGF actions in CNS: implications in multiple sclerosis. Cytokine Growth Factor Rev. 2007;18(3–4):267–278. 17485236 10.1016/j.cytogfr.2007.04.001

[26] Streit WJ, Walter SA, Pennell NA. Reactive microgliosis. Prog Neurobiol. 1999;57(6):563–581. 10221782 10.1016/s0301-0082(98)00069-0

[27] Klempt ND, Klempt M, Gunn AJ, et al. Expression of insulin-like growth factor-binding protein 2 (IGF-BP 2) following transient hypoxia-ischemia in the infant rat brain. Brain Res Mol Brain Res. 1992;15(1–2):55–61. 1279350 10.1016/0169-328x(92)90151-z

[28] Baker NL, Carlo Russo V, Bernard O, et al. Interactions between bcl-2 and the IGF system control apoptosis in the developing mouse brain. Brain Res Dev Brain Res. 1999;118(1–2):109–118. 10611509 10.1016/s0165-3806(99)00136-4

